# HIF-2α dictates the susceptibility of pancreatic cancer cells to TRAIL by regulating survivin expression

**DOI:** 10.18632/oncotarget.17157

**Published:** 2017-04-17

**Authors:** Nanae Harashima, Keizo Takenaga, Miho Akimoto, Mamoru Harada

**Affiliations:** ^1^ Department of Immunology, Shimane University Faculty of Medicine, Shimane, Japan; ^2^ Department of Life Science, Shimane University Faculty of Medicine, Shimane, Japan

**Keywords:** hypoxia, HIF-2α, TRAIL, surviving, pancreatic cancer

## Abstract

Cancer cells develop resistance to therapy by adapting to hypoxic microenvironments, and hypoxia-inducible factors (HIFs) play crucial roles in this process. We investigated the roles of HIF-1α and HIF-2α in cancer cell death induced by tumor necrosis factor (TNF)-related apoptosis-inducing ligand (TRAIL) using human pancreatic cancer cell lines. siRNA-mediated knockdown of HIF-2α, but not HIF-1α, increased susceptibility of two pancreatic cancer cell lines, Panc-1 and AsPC-1, to TRAIL *in vitro* under normoxic and hypoxic conditions. The enhanced sensitivity to TRAIL was also observed *in vivo*. This *in vitro* increased TRAIL sensitivity was observed in other three pancreatic cancer cell lines. An array assay of apoptosis-related proteins showed that knockdown of HIF-2α decreased survivin expression. Additionally, survivin promoter activity was decreased in HIF-2α knockdown Panc-1 cells and HIF-2α bound to the hypoxia-responsive element in the survivin promoter region. Conversely, forced expression of the *survivin* gene in HIF-2α shRNA-expressing Panc-1 cells increased resistance to TRAIL. In a xenograft mouse model, the survivin suppressant YM155 sensitized Panc-1 cells to TRAIL. Collectively, our results indicate that HIF-2α dictates the susceptibility of human pancreatic cancer cell lines, Panc-1 and AsPC-1, to TRAIL by regulating survivin expression transcriptionally, and that survivin could be a promising target to augment the therapeutic efficacy of death receptor-targeting anti-cancer therapy.

## INTRODUCTION

Hypoxia is an important condition for solid tumors to progress and metastasize. The concentration of oxygen in normal tissues is approximately 5%, whereas in solid tumors is usually below 1% [1]. Hypoxia leads to cellular responses involving stabilization of hypoxia-inducible factors (HIFs), which are transcription factors composed of an inducible α-subunit (HIFs-1α–3α) and a constitutive β-subunit [2]. Under normoxic conditions, HIF-α proteins are degraded through the ubiquitin-proteasome pathway [3], whereas the HIF-α and β-subunit complexes accumulate under hypoxic conditions. Subsequently, these complexes translocate to the nucleus and bind to hypoxia-responsive elements (HREs) in the promoter regions of targeted genes. Although HIFs are inducible in normal cells [4, 5], their expressions are frequently higher in various types of cancers [6]. A representative example is renal cell carcinoma (RCC), in which HIF is highly expressed due to frequent mutations of the *von Hippel-Lindau* (*VHL*) gene [7]. Pancreatic cancers are poorly vascularized and thus very hypoxic. In addition, HIFs play critical roles in cancer cells resistant to therapy [8, 9]. Although extensive studies have been performed on HIFs, they have mainly focused on HIF-1α. Accordingly, the roles of HIF-2α in cancers resistant to therapy have not been fully investigated.

Immune cells lyse cancer cells via perforin/granzyme, Fas ligand, tumor necrosis factor (TNF)-α, and TNF-related apoptosis-inducing ligand (TRAIL). Among these, TRAIL binds to death receptors (DRs) on cancer cells and triggers apoptosis while sparing normal cells [10, 11]. DR4 (TRAIL-R1) and DR5 (TRAIL-R2) belong to the TNF receptor gene superfamily, all of which share a similar, cysteine-rich extracellular domain and an additional cytoplasmic death domain [12]. TRAIL also binds to two decoy receptors, DcR1 (TRAIL-R3) and DcR2 (TRAIL-R4). These DcRs lack the cytoplasmic signaling components required for the transmission of the apoptosis signaling [13]. When TRAIL binds to DR4 and DR5, caspase-8 is recruited to a death-inducing signaling complex. Activation of caspase-8 induces ‘extrinsic’ apoptosis, as well as mitochondria-mediated ‘intrinsic’ apoptosis via activation of caspase-9. Recombinant TRAIL and anti-DR agonistic antibodies have attracted attention as potential therapeutics for treating various malignancies, although the results of clinical trials have been unsatisfactory. This has led several researchers to propose that cancer cells gain resistance via various possible mechanisms, such as downregulation of DRs and/or upregulation of anti-apoptotic proteins including cellular FLICE-like inhibitory protein (c-FLIP), the Bcl-2 family of proteins, and inhibitors of the apoptosis protein family [14, 15]. Additionally, sensitivity of a panel of human pancreatic cancer cell lines to TRAIL is inversely correlated with their Bcl-xL expression [16].

In this study, we investigated the roles of HIF-1α and HIF-2α in TRAIL-induced human pancreatic cancer cell death. We found for the first time that HIF-2α dictates the susceptibility of pancreatic cancer cell lines, Panc-1 and AsPC-1, to TRAIL, by transcriptionally regulating survivin, an anti-apoptotic molecule, and that survivin may be a promising target to augment the therapeutic efficacy of DR-targeting anti-cancer therapy.

## RESULTS

### siRNA-mediated knockdown of HIF-2α increases TRAIL sensitivity of pancreatic cancer cells

**Figure 1:**
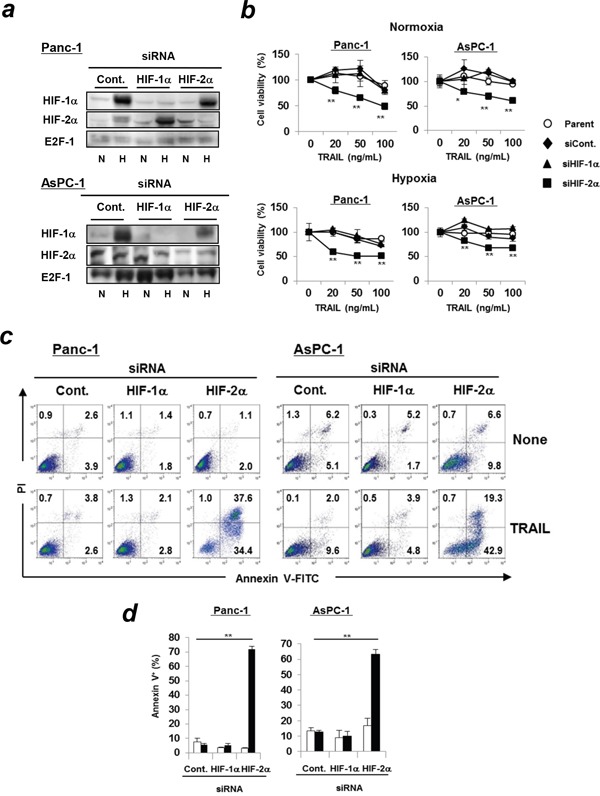
Transfection of HIF-2α siRNA increases TRAIL sensitivity of pancreatic cancer cells **(a)** Panc-1 and AsPC-1 cells were transfected with the indicated siRNAs and cultured under hypoxic (1% O_2_) or normoxic conditions for 24 h, and then subjected to immunoblotting using nuclear extracts. E2F-1 was used as a loading control. N: normoxic, H: hypoxic. **(b)** siRNA-transfected cancer cells were cultured with TRAIL under normoxic or hypoxic conditions for 48 h. Cell viability was analyzed using the WST-8 assay. Similar results were obtained from two independent experiments. *p < 0.05, **p < 0.01 were compared to negative control siRNA. **(c)** Two cell lines were incubated with TRAIL (100 ng/mL, 24 h for Panc-1 or 50 ng/mL, 12 h for AsPC-1) under normoxic conditions and flow cytometric analysis was performed. Similar results were obtained from two independent experiments. **(d)** The results are presented as means ± SD from triplicate experiments. The open and closed bars represent the data of control and TRAILtreatment, respectively. ** *p* < 0.01.

We first confirmed the expression status of HIFs and the efficiency of siRNA-mediated knockdown (Figure 1a). Immunoblotting was performed using the nuclear lysates because detection of HIFs in whole lysates was difficult. In terms of Panc-1 cells, both HIFs were expressed at low levels even under normoxic conditions. Hypoxia increased their expression and the siRNA-mediated knockdown was successful. Conversely, HIF-2α was clearly detected in the nuclear lysate of AsPC-1 cells under normoxic condition and siRNA transfection decreased their expression. Next, we examined the sensitivity of these cell lines to TRAIL and found that siRNA-mediated knockdown of HIF-2α, but not HIF-1α, significantly decreased the cell viability of the TRAIL-treated cell lines under normoxic and hypoxic conditions (Figure 1b). In addition, the percentages of Annexin V^+^ apoptotic cells were significantly increased in HIF-2α siRNA-transfected cancer cells under normoxic (Figure 1c and 1d) and hypoxic conditions (Supplementary Figure 1a and 1b). We also examined the DR5 expression on Panc-1 and AsPC-1 cells transfected with control, HIF-1α, or HIF-2α siRNA and found that knockdown of HIF-1α tended to decrease the DR5 expression on both cell lines (Supplementary Figure 2). However, DR5 expression was unchanged on HIF-2α siRNA-transfected cancer cell lines under normoxic and hypoxic conditions.

### Caspase-dependent apoptosis in HIF-2α siRNA-transfected and TRAIL-treated Panc-1 cells

**Figure 2:**
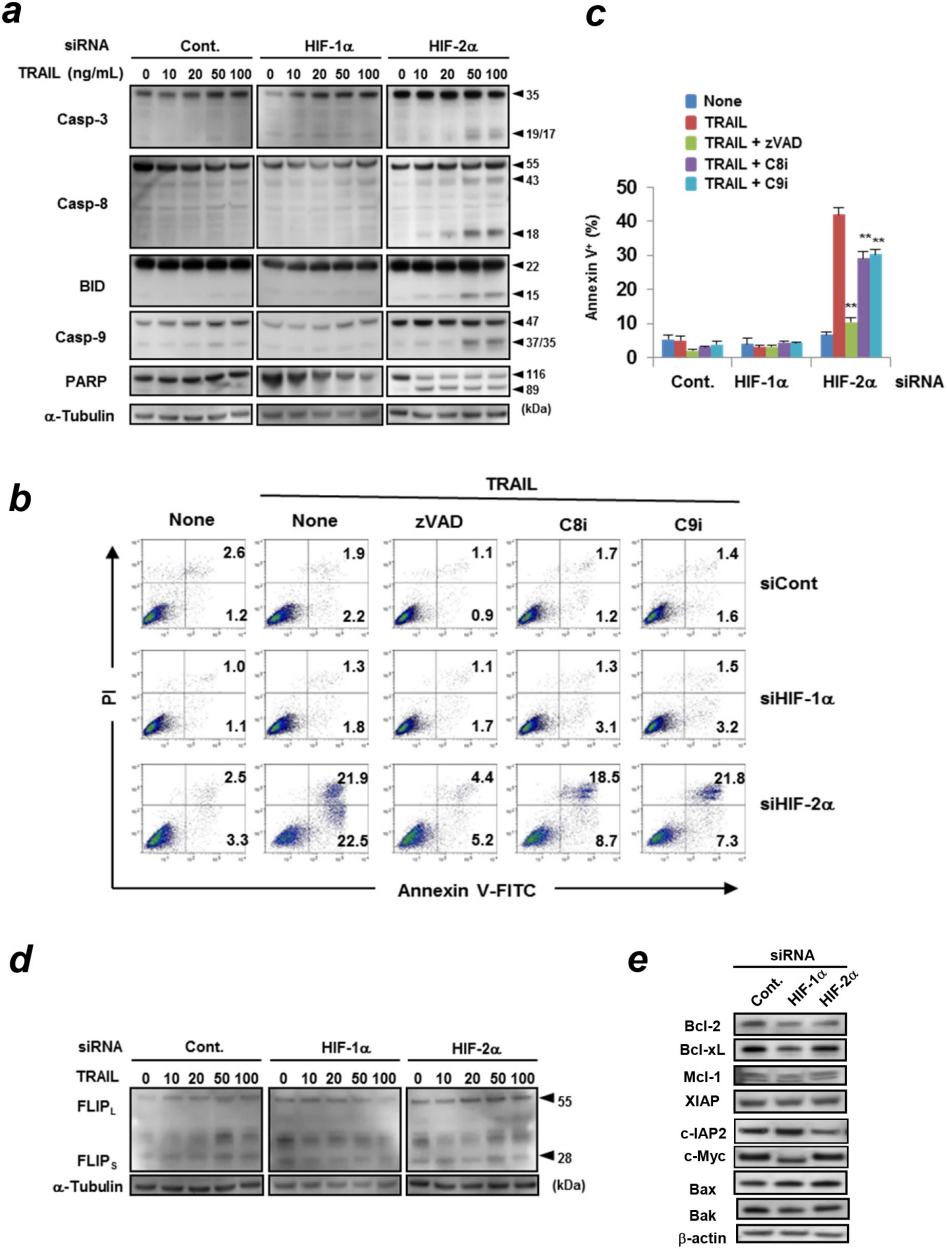
Caspase-dependent apoptosis of HIF-2α siRNA-transfected and TRAIL-treated Panc-1 cells **(a)** siRNA transfected Panc-1 cells were treated with various doses of TRAIL for 6 h. Protein lysates from whole cells were assayed using immunoblotting. α-tubulin was used as a loading control. **(b)** siRNA transfected Panc-1 cells were cultured with TRAIL (100 ng/mL) in the presence of the indicated caspase inhibitors (20 μM). The cells were examined using flow cytometric analysis. The number represents the percentages of each subset. zVAD, pan-caspase inhibitor (z-VAD-FMK); C8i, caspase-8 inhibitor (z-IETD-FMK); C9i, caspase-9 inhibitor (z-LEHD-FMK). Similar results were obtained from two independent experiments. **(c)** The results of Annexin V^+^ cells (%) are presented as means ± SD from triplicate experiments. **p < 0.01. **(d)** siRNA transfected Panc-1 cells were treated with the indicated concentration of TRAIL for 6 h. The protein expression levels of c-FLIP were determined using immunoblotting. α-tubulin was used as a loading control. **(e)** Similarly, the lysates were used for immunoblotting to examine the expression of the indicated proteins. β-actin was used as a loading control.

Next, we investigated the involvement of caspases in apoptosis of HIF-2α siRNA-transfected and TRAIL-treated Panc-1 cells. Knockdown of HIF-2α increased the expression of cleaved caspase-3, -8, -9, and poly (ADP-ribose) polymerase (PARP) in TRAIL-treated Panc-1 cells in a dose-dependent manner (Figure 2a). Bid, a BH-3 domain-only protein, connects the extrinsic and intrinsic apoptosis pathways. The expression of truncated Bid was increased in HIF-2α siRNA-transfected and TRAIL-treated Panc-1 cells. Additionally, the addition of z-VAD-FMK, a pan-caspase inhibitor, significantly inhibited apoptosis of HIF-2α siRNA-transfected and TRAIL-treated Panc-1 cells (Figure 2b and 2c). The addition of either caspase-8 or caspase-9 inhibitor partially but significantly decreased the percentages of apoptosis. We also examined the expression of c-FLIP and several anti-apoptotic proteins, including those in the Bcl-2 family or inhibitors of apoptosis (IAP) family, which are often involved in resistance to TRAIL. No changes in the expression of either c-FLIP_L_ or c-FLIP_S_ were observed (Figure 2d). In addition, knockdown of HIF-2α did not affect the expression of anti-apoptotic proteins (Bcl-2, Bcl-xL, and Mcl-1), a member of the IAP family (XAIP) and pro-apoptotic proteins (Bax and Bak) in Panc-1 cells (Figure 2e). We also examined the expression of c-IAP2 and c-Myc in siRNA-transfected Panc-1 cells. The results showed that knockdown of HIF-1α increased the expression of c-IAP2, while HIF-2α knockdown decreased expression. In addition, knockdown of HIF-1α decreased the expression of c-Myc.

### The varied roles of HIF-1α and HIF-2α in the TRAIL sensitivity of other cancer cell lines

**Figure 3:**
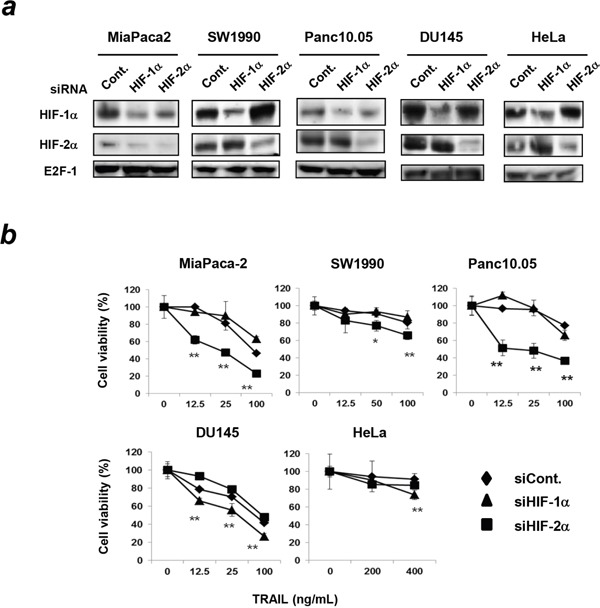
Effect of knockdown of either HIF-1α or HIF-2α on TRAIL sensitivity in other cancer cell lines **(a)** Three pancreatic cancer cell lines (MiaPaca-2, SW1990, and Panc10.05), a prostate cancer cell line (DU145), and a cervical cancer cell line (HeLa) were transfected with the indicated siRNAs and cultured under hypoxic conditions (1% O_2_) for 24 h. Then, nuclear extracts of the cells were subjected to immunoblotting. E2F-1 was used as a loading control. **(b)** siRNA-transfected cancer cells were cultured with TRAIL under hypoxic conditions for 48 h. Cell viability was analyzed using the WST-8 assay. The results are presented as means ± SD from triplicate experiments. *p < 0.05, **p < 0.01 compared to negative control siRNA.

We further examined the TRAIL sensitivity of other pancreatic cancer cell lines and of cell lines of other cancer types under hypoxic conditions. siRNA-mediated knockdown of HIF-1α or HIF-2α selectively decreased expression of the respective protein in the three pancreatic cancer cell lines MiaPaca-2, SW1990, and Panc10.05; in the prostate cancer cell line DU145; and in the cervical cancer cell line HeLa (Figure 3a). Next, we examined the sensitivity of these cell lines to TRAIL and found that siRNA-mediated knockdown of HIF-2α, but not HIF-1α, significantly decreased the cell viability of the TRAIL-treated three pancreatic cancer cell lines under hypoxic conditions (Figure 3b). However, knockdown of HIF-1α or HIF-2α did not affect the TRAIL sensitivity of two other human pancreatic cancer cell lines, CAPAN-2 and CFPAC-1 (data not shown). As a whole, knockdown of HIF-2α increased TRAIL sensitivity of five out of the seven human pancreatic cancer cell lines evaluated. Alternatively, although the difference was small, siRNA-mediated knockdown of HIF-1α, but not HIF-2α, increased TRAIL sensitivity in a prostate (DU145) and a cervical cancer cell line (HeLa) (Figure 3b).

### A protective role of HIF-2α in apoptosis of TRAIL-treated Panc-1 cells *in vitro* and *in vivo*

**Figure 4:**
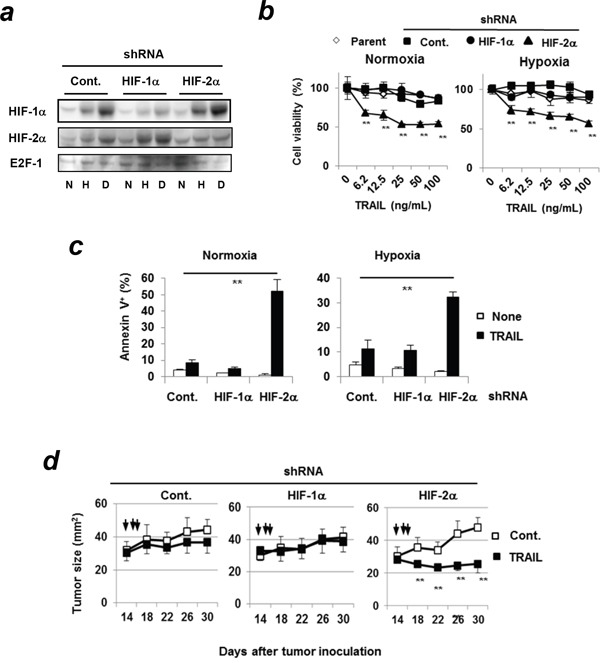
TRAIL sensitivity of HIF-2α shRNA-expressing Panc-1cells both *in vitro* and *in vivo* **(a)** The nuclear extracts of Panc-1 cells were used for immunoblotting. E2F-1 was used as a loading control. N: normoxic, H: hypoxic (1% O_2_ for 24 h), D: deferoxamine mesylate (100μM, normoxic for 24 h). **(b)** shRNA-expressing Panc-1 cells were treated with TRAIL at the indicated concentrations under hypoxia or normoxia for 48 h. Cell viability was assessed using the WST-8 assay. **p < 0.01 was compared to control shRNA-transduced cells. Similar results were obtained from three independent experiments. **(c)** shRNA-expressing Panc-1 cells were incubated with 100 ng/mL TRAIL for 24 h and analyzed using flow cytometry. The results of Annexin V^+^ cells (%) are presented as means ± SD from triplicate experiments. **p < 0.01 **(d)** BALB/c *nu/nu* mice were inoculated with the indicated Panc-1 cells (5 × 10^6^) with Matrigel into the right upper and lower two sites. On days 14, 15, and 16, vehicle (upper) control and TRAIL (1 μg; lower) at a volume of 50 μL were injected into the tumor sites, respectively. Each group consisted of six mice. Similar results were obtained from two independent experiments. **p < 0.01.

To further investigate the roles of HIFs in apoptosis of TRAIL-treated Panc-1 cells, shRNA-expressing Panc-1 cell lines were established. HIF-1α shRNA or HIF-2α shRNA-transduced Panc-1 cells decreased the expression of HIF-1α and HIF-2α, respectively (Figure 4a). Deferoxamine mesylate, an iron chelator, was used as a hypoxia-mimetic agent. This agent increased the expression of HIFs similarly with hypoxic conditions. As observed in the siRNA-transfected Panc-1 cells (Figure 1b), HIF-2α shRNA-transduced Panc-1 cells increased their sensitivity to TRAIL (Figure 4b) and the percentages of Annexin V^+^ apoptotic cells were increased only in HIF-2α shRNA-expressing Panc-1 cells under normoxic and hypoxic conditions (Figure 4c, Supplementary Figure 3). In a xenograft mouse model, HIF-2α shRNA-expressing Panc-1 cells were more susceptible to TRAIL *in vivo* compared to the other groups (Figure 4d).

### Contribution of survivin to HIF-2α-associated resistance of Panc-1 cells to TRAIL

**Figure 5:**
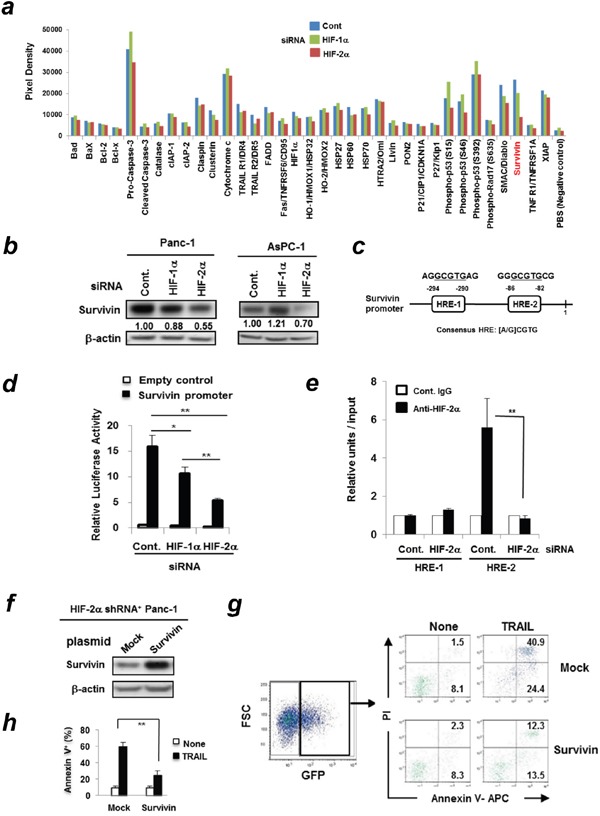
HIF-2α dictates TRAIL sensitivity of Panc-1 cells by regulating survivin expression **(a)** Panc-1 cells were transfected with control, HIF-1α, or HIF-2α siRNA and the whole cell protein was extracted. Equal amounts of protein (500 μg) were used for the Proteome Profiler Apoptosis Array. **(b)** Panc-1 and AsPC-1 cells were transfected with the indicated siRNA. After 72 h, the cell lysates were used for immunoblotting. The band density of survivin was normalized to β-actin. **(c)** Two HREs in the human survivin promoter are shown. **(d)** Panc-1 cells were pre-transfected with either control HIF-1α or HIF-2α siRNA. On the next day, cells were co-transfected with TransLucent Survivin Gene Promoter Reporter Vector and pGL4.74 (hRluc/TK) vector. After 24 h incubation, the ratio of firefly/renilla luciferase was determined. The results are presented as means ± SD from triplicate experiments. Similar results were obtained from three independent experiments. *p < 0.05, **p < 0.01. **(e)** ChIP assay was performed on Panc-1 cells transfected with siRNA against HIF-2α or control. Data represents means ± SD from triplicate results which were normalized to input DNA. *, *p* < 0.05. **(f)** Immunoblotting was performed using the protein lysates from HIF-2α shRNA-expressing Panc-1 cells that were pre-transfected with mock control or survivin plasmid. **(g)** HIF-2α shRNA-expressing Panc-1 cells transfected with GFP-encoding mock or survivin plasmid were treated with TRAIL (100 ng/mL) for 24 h. Flow cytometric analysis was performed. After gating on survivin-GFP^+^ cells, the percentages of Annexin V^+^ apoptotic cells were determined. The numbers represent the percentages of each susbset. Similar results were obtained from two independent experiments. **(h)** The results of Annexin V^+^ cells (%) are presented as means ± SD from triplicate experiments. **p < 0.01.

To determine the mechanisms underlying the increased sensitivity of HIF-2α-knocked-down Panc-1 cells to TRAIL, we examined changes in a panel of apoptosis-associated molecules using the Proteome Profiler Human Apoptosis Array. The cell lysates were prepared from Panc-1 cells pretransfected with either control, HIF-1α, or HIF-2α siRNA. The results showed that survivin, a member of the IAP family, was reduced in HIF-2α siRNA-transfected Panc-1 cells (Figure 5a, Supplementary Figure 4a). No definite changes were observed in the other IAP family members such as cIAP and XIAP. In addition, siRNA-mediated knockdown of HIF-2α markedly decreased the protein expression of survivin in Panc-1 and AsPC-1 cell lines (Figure 5b).

Next, we searched for the potential HIF-2α-binding sites in the proximal promoter of the human *survivin* gene (BIRC5, NM_001168) and found two hypoxia-responsive elements (HREs) containing the consensus sequence (A/G) CGTG (Figure 5c). We also compared the survivin promoter activity of Panc-1 cells, which were pre-transfected with either control, HIF-1α, or HIF-2α siRNA. The HIF-2α siRNA-transfected Panc-1 cells showed significantly decreased survivin reporter activity compared to the other groups (Figure 5d). The survivin reporter activity was decreased in HIF-1α siRNA-transfected Panc-1 cells; the decrease was almost half that of HIF-2α siRNA-transfected Panc-1 cells. We further determined which HRE sites HIF-2α bound to using ChIP assay (Figure 5e). As a result, HIF-2α preferentially bound to the HRE-2 in the survivin promoter region and this binding was abolished in HIF-2α siRNA-transfected Panc-1 cells.

Next, we confirmed that transfection of the *survivin* gene-encoding plasmid into HIF-2α shRNA-expressing Panc-1 cells increased the expression of survivin protein (Figure 5f). Because the *survivin* gene-encoding plasmid also contained the *GFP* gene, we could discriminate survivin-expressing GFP^+^ Panc-1 cells from survivin-negative GFP^−^ Panc-1 cells. As shown in Figure 5g and 5h, the forced expression of survivin protein rendered HIF-2α shRNA-expressing Panc-1 cells more resistant to TRAIL compared to the mock control group.

### The survivin suppressant YM155 sensitizes Panc-1 cells to TRAIL both *in vitro* and *in vivo*

**Figure 6:**
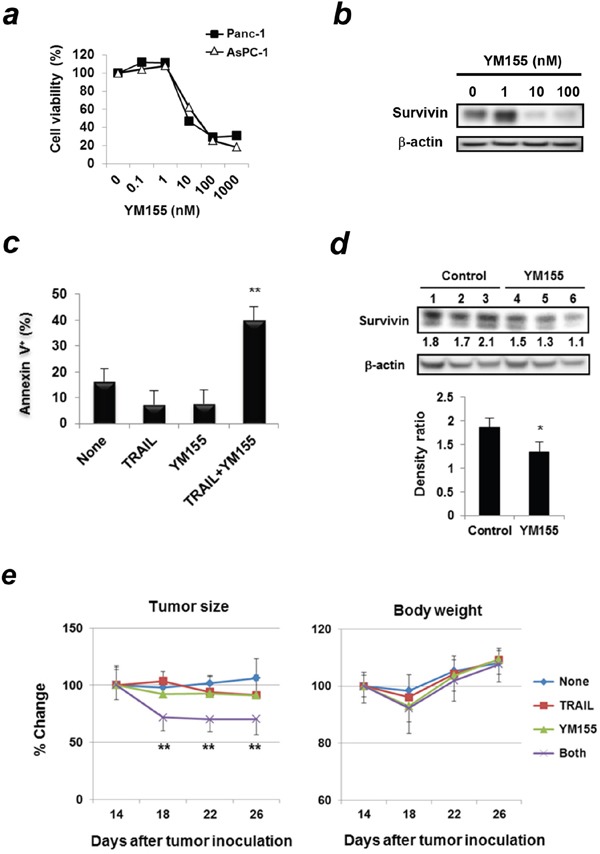
Survivin suppressant YM155 sensitizes Panc-1 cells to TRAIL both *in vitro* and *in vivo* **(a)** Two cancer cell lines were treated with YM155 for 48 h. Cell viability was assessed using the WST-8 assay. Similar results were obtained from two independent experiments. **(b)** Whole cell lysates from Panc-1 cells treated with YM155 for 24 h were used for immunoblotting. β-actin was used as a control. **(c)** Panc-1 cells treated with YM155 (5 nM) and/or TRAIL (25 ng/mL) were examined using flow cytometric analysis. Results of Annexin V^+^ cells (%) are presented as means ± SD from triplicate experiments. **p < 0.01 was compared to untreated control. Similar results were obtained from two independent experiments. **(d)** BALB/c *nu/nu* mice were inoculated s.c. with Panc-1 cells (5 × 10^6^) with Matrigel into the right flank; the mice were pooled and divided into 2 groups. The mice were injected intraperitoneally (i.p.) with YM155 (5 mg/kg) or DMSO as a vehicle control (50 μL) for consecutive 3 days. Each group contained three mice. Protein lysates from tumor tissues were used for immunoblotting. The band density of survivin was normalized to β-actin. *p < 0.05. **(e)** Similarly, BALB/c *nu/nu* mice were inoculated subcutaneously with Panc-1 cells (5 × 10^6^). On day 14, the mice were pooled and divided into four groups. The mice were treated with i.p. injection of YM155 (5 mg/kg) or DMSO as a vehicle control (50 μL) on days 14, 15, and 16. Each group contained six mice. After grouping, mice were treated with i.p. injection of YM155 (5 mg/kg) for 5 consecutive days (on days 14, 15, 16, 17, and 18) and with or without i.t. injection of TRAIL (1 μg) on days 16 and 18. Each group consisted of six mice. **p < 0.01 compared to vehicle control group. Similar results were obtained from two independent experiments.

The aforementioned results suggest that HIF-2α controls sensitivity to TRAIL by regulating survivin expression. Therefore, we tested if inhibition of survivin could increase the sensitivity of human pancreatic cancer cells to TRAIL using YM155, a novel survivin suppressant used clinically [17, 18]. YM155 reduced the cell viability of two pancreatic cancer cell lines in a dose-dependent manner (Figure 6a) and decreased the survivin protein levels in Panc-1 cells (Figure 6b). Interestingly, the combination of TRAIL and YM155 increased the percentages of Annexin V^+^ apoptotic cells compared to treatment with either alone (Figure 6c, and Supplementary Figure 4b). We further examined the *in vivo* effect of YM155 using a xenograft mouse model. The *in vivo* treatment with YM155 significantly decreased survivin protein expression in Panc-1 cells that were subcutaneously (s.c.) established in nude mice compared to the vehicle control (Figure 6d). Lastly, we examined the combined effect of TRAIL and YM155 on Panc-1 *in vivo*. The combination of TRAIL and YM155 significantly suppressed the growth of Panc-1 compared to the treatment with either alone (Figure 6e). Although the YM155 treatment alone or the combination of YM155 and TRAIL tended to decrease body weight, these changes were transient and insignificant.

## DISCUSSION

Hypoxia renders cancer cells resistant to therapy and HIFs play central roles in this process [19]. Therefore, the HIF-hypoxia system is a promising target for the development of novel anti-cancer therapies [20]. Despite the high homology between HIF-1α and HIF-2α, these two molecules conversely regulate key downstream genes. HIF-1α decreases c-Myc, mTOR, and β-catenin and increases p53 activity, whereas HIF-2α has the opposite effects [21]. To date, studies on HIF-2α in cancer cell death have frequently been performed with those of HIF-1α. One study reported that sorafenib, a multikinase inhibitor, switches from HIF-1α- to HIF-2α-dependent pathways, resulting in resistance to sorafenib in hypoxic hepatocellular carcinoma (HCC) by activating the TGF-α/EGFR pathway [22]. In RCC, overexpression of HIF-2α increases tumor growth, whereas HIF-1α shows a reverse effect [23]. HIF-2α, but not HIF-1α, upregulates the expression of the *Snail1* gene and induces resistance to temozolomide and cisplatin [24]. Some other studies have focused on HIF-2α. Several clinical studies have demonstrated that high levels of HIF-2α are well correlated with advanced clinical stage and poor prognosis in neuroblastoma [25]. Conversely, other studies have shown pro-apoptotic roles of HIF-2α in cancer cell death. shRNA-mediated knockdown of HIF-2α inhibits TRAIL-induced apoptosis in RCC by decreasing the DR5 level at the transcriptional level [26]. In our study, knockdown of HIF-2α in pancreatic cancer cells did not affect DR5 expression (Supplementary Figure 2). A previous report indicated that knockdown of HIF-1α in HCC enhances HIF-2α expression and vice versa, and that, in both cases, the expression of anti-apoptotic Bcl-xL was increased, resulting in attenuated apoptosis and enhanced autophagy [27]. Regarding Bcl-xL and sensitivity to TRAIL, we recently revealed that sensitivity of a panel of pancreatic cancer cell lines to TRAIL was inversely correlated with their Bcl-xL expression [16]. However, in this study, knockdown of HIF-2α in Panc-1 cells showed no effect on Bcl-xL expression; it also did not affect the expression of c-FLIP (Figure 2d), or anti-apoptotic proteins (Bcl-2, Bcl-xL, and Mcl-1) (Figure 2e).

Most studies have focused on HIF-1α when examining roles of HIFs in TRAIL-induced cancer cell death. Knockdown of HIF-1α can sensitize human colon cancer, neuroblastoma, uterine cervical cancer, lung cancer, and gastric cancer cells to TRAIL-mediated apoptosis [28, 29]. Since a previous report suggested that c-Myc and c-IAP2 play positive and negative roles in TRAIL-induced apoptosis of hypoxic human colon cancer cells, respectively [28], we examined the expression of these molecules in siRNA-transfected Panc-1 cells. Knockdown of HIF-1α increased the expression of c-IAP2 but decreased the expression of c-Myc, whereas knockdown of HIF-2α decreased the expression of c-IAP2 (Figure 2e). The similar result was observed in the apoptosis array assay (Figure 5a). The decreased expression of c-IAP2 in HIF-2α-knockdown Panc-1 cells may partially explain why TRAIL-induced apoptosis was induced in HIF-2α-knockdown cells, but not in HIF-1α-knockdown cells. In this study, we examined the effects of HIFs on the TRAIL sensitivity of a panel of human pancreatic cancer cell lines and found that knockdown of HIF-2α, but not HIF-1α, increased TRAIL sensitivity in five out of the seven pancreatic cancer cell lines evaluated. However, many other studies have indicated that knockdown of HIF-1α sensitizes various types of human cancer cell types to apoptosis [28, 29]. Actually, we observed that knockdown of HIF-1α, but not HIF-2α, increased the TRAIL sensitivity of a prostate (DU145) and a cervical cancer cell line (HeLa) (Figure 3b). These results suggest that there are differences in the roles of HIF-1α and HIF-2α that are specific to each cancer type in terms of TRAIL sensitivity. Further studies are needed to elucidate the precise mechanisms.

To determine the regulatory role of HIF-2α in the TRAIL-induced apoptosis of pancreatic cancer cells, we examined changes in apoptosis-related molecules using the Proteome Profiler Human Apoptosis Array. Survivin was decreased in HIF-2α-knocked-down Panc-1 cells (Figure 5a). In addition, the protein expression of survivin was decreased in HIF-2α siRNA-transfected pancreatic cancer cell lines (Figure 5b) and the forced *survivin* gene expression in HIF-2α shRNA-expressing Panc-1 cells increased their resistance to TRAIL (Figure 5g and 5h). Survivin is a member of the IAP family that is highly expressed in pancreatic cancer tissues but not in normal pancreatic tissues [30] and has been implicated in both suppression of apoptosis and regulation of mitosis [31]. Survivin is expressed at low levels or undetected in normal cells but highly expressed in many types of tumors [32, 33]. Therefore, survivin can be considered a tumor antigen for anti-cancer immunotherapy [34, 35]. To date, several studies have suggested a relationship between HIF-1α and survivin in several types of cancers. In contrast, only one report has suggested an association between HIF-2α and survivin in gastric cancer [36].

Because the two HREs contained the consensus sequence in the promoter region of the *survivin* gene, we performed the survivin promoter reporter assay and found that survivin reporter activity decreased in HIF-2α siRNA-transfected Panc-1 cells (Figure 5d). ChIP assay revealed that HIF-2α bound to the HRE-2 in the survivin promoter (Figure 5e). These results indicate that HIF-2α regulates the survivin expression in Panc-1 cells at the transcriptional level. Knockdown of HIF-1α decreased the survivin reporter activity level by half compared to knockdown of HIF-2α. HIF-1α could bind to HREs in the promoter region of the *survivin* gene. Actually, a previous study has shown that the expression of survivin was positively correlated with HIF-1α[37]. Furthermore, EGF-signaling induced HIF-1α expression and HIF-1α is directly bound to HRE site, which corresponds to the HRE-2 in Figure 5c, in the survivin core promoter [38]. Nevertheless, in this study, HIF-2α appears to play more important roles in the regulation of survivin expression than HIF-1α. We presently have no clear explanation on this issue.

Because survivin plays an anti-apoptotic role in cancer cells, it may be a promising target as a therapeutic for anti-cancer treatment. Survivin inhibition has been shown to sensitize cancer cells to several chemotherapeutic drugs both *in vitro* and *in vivo* [39, 40]. YM155 is a survivin suppressant and can suppress its expression in diverse human cancer cells. Xenograft models have revealed antitumor activity of YM155 alone or in combination with docetaxel [41]. Other reports have also demonstrated antitumor activity of YM155 alone or in combination with various anticancer drugs [42, 43]. YM155 alone or in combination has been used in clinical trials against various types of malignancies, including lymphomas, melanomas, prostate, lung, and colorectal cancers [17, 18, 44]. We showed that YM155 significantly augmented TRAIL-induced apoptosis in pancreatic cancer cells both *in vitro* and *in vivo* (Figure 6c and 6e).

In conclusions, we demonstrated for the first time that HIF-2α dictates the resistance of human pancreatic cancer cells to TRAIL under normoxic and hypoxic conditions and transcriptionally regulates survivin expression. We also showed that a survivin suppressant, YM155, can sensitize human pancreatic cancer cells to TRAIL both *in vitro* and *in vivo*. In addition to its tumor antigen capabilities, survivin could be a target candidate to augment the therapeutic efficacy of DR-targeting anti-cancer therapy.

## MATERIALS AND METHODS

### Cell cultures

Panc-1 and MiaPaca-2 are human pancreatic cancer cell lines [45]. Other human pancreatic cancer cell lines (AsPC-1, SW1990, and Panc10.05) were purchased from the ATCC. The human cervical cancer cell line HeLa was kindly provided by Dr. H. Uemura (Kinki University). All of the above cell lines were maintained in DMEM (Nacalai Tesque) supplemented with 10% fetal bovine serum (Sigma-Aldrich), 1 mM sodium pyruvate (Nacalai Tesque), and 20 μg/mL gentamicin (Nacalai Tesque). The human prostate cancer cell line DU145 was obtained from the ATCC and was maintained in RPMI-1640 medium (Sigma-Aldrich) supplemented with 10% fetal bovine serum (Sigma-Aldrich) and 20 μg/ml gentamicin. All cells were maintained at 37°C in a 5% CO_2_ incubator. For hypoxic culture, cells were incubated under 1% O_2_/94% N_2_/5% CO_2_ conditions in a humidified automatic O_2_/CO_2_ incubator (Wakenyaku).

### Reagents

Recombinant human TRAIL and IL-2 were purchased from PeproTech. YM155 (sepantronium bromide) was purchased from Cayman Chemical. Deferoxamine mesylate was purchased from Wako Chemical. The following caspase inhibitors were added to the *in vitro* proliferation assay 2 h before adding TRAIL at a dose of 20 μM: pan-caspase inhibitor z-VAD-FMK (Enzo Life Sciences), caspase-8 inhibitor z-IETD-FMK (R&D Systems), and caspase-9 inhibitor z-LEHD-FMK (R&D Systems).

### Cell viability assay

Cell viability was analyzed using the WST-8 assay (Nakalai Tesque). Briefly, cells were seeded into 96-well flat-bottom plates, 10 μL WST-8 solution was added to each well at the end of the incubation, and the plates were incubated for an additional 3 h. Absorbance in each well was measured at 450 nm using a microplate reader (Beckman Coulter).

### siRNA transfection

siRNA transfection was performed using the Lipofectamine RNAiMAX transfection reagent (Invitrogen) according to the manufacturer's instructions. The following siRNAs were used: HIF-1α siRNA (Santa Cruz Biotechnology [SCB]), HIF-2α siRNA (Invitrogen), survivin siRNA II (Cell Signaling Technology [CST]), and Silencer select negative control No. 1 siRNA (Ambion). Cells were used for experiments 3 days after siRNA transfection.

### Establishment of shRNA-expressing cancer cell lines

For stable knockdown of HIF-1α or HIF-2α, MISSION human HIF-1α shRNA or human HIF-2α shRNA lentiviral vectors in the pLKO.1-puro plasmid (Sigma-Aldrich) were used. For the controls, MISSION pLKO.1-puro control transduction particles (SHC001v) were used. Panc-1 cells were transduced with the lentivirus stocks in the presence of Polybrene (8 μg/ml) and then selected with puromycin (5 μg/ml) to allow for the generation of the control cells (shCont) or of cells displaying stable HIF-1α (shHIF-1α) or HIF-2α (shHIF-2α) downregulation.

### Apoptosis assay

Apoptosis was examined using the Annexin V-FITC Apoptosis Detection Kit (BioVision) according to the manufacturer's instructions. In brief, cells were stained with FITC-conjugated or APC-conjugated Annexin V and PI. Stained cells were analyzed using FACSCalibur (BD Biosciences). Data were plotted using Flowlogic^TM^ software (Inivai).

### Immunoblotting

Whole cell lysates were extracted using M-PER reagent (Thermo Fisher Scientific) with protease inhibitor cocktail (Nacalai Tesque). To detect HIFs, nuclear extracts from cultured cells were separated using NE-PER nuclear and cytoplasmic extraction reagents (Thermo Fisher Scientific). To analyze protein expression in tissue samples from mice, T-PER tissue protein extraction reagent (Thermo Fisher Scientific) was used. NuPAGE Novex Bis-Tris gels (4–12% or 12%; Thermo Fisher Scientific) were used for protein separation, and the proteins were immobilized onto PVDF membranes (Thermo Fisher Scientific) using the iBlot transfer system (Thermo Fisher Scientific). The membranes were incubated with the following primary antibodies: anti-HIF-1α (BD Transduction Laboratories), anti-HIF-2α (NOVUS Biologicals), anti-Bid (CST), anti-caspase-3 (CST), anti-PARP (CST), anti-Bax (CST), anti-Bak (CST), anti-survivin (CST), anti-XIAP (CST), anti-c-IAP2 (CST), anti-c-Myc (Epitomics), anti-Bcl-2 (CSB), anti-FLIP _S/L_ (SCB), anti-Mcl-1 (SCB), anti-E2F-1 (SCB), anti-α-tubulin (SCB), anti-caspase-8 (Medical and Biological Laboratorie [MBL]), anti-caspase-9 (MBL), anti-Bcl-X_S/L_ (Biolegend), and anti-β-actin (Biolegend). Protein bands were visualized using AP Chemiluminescent Substrate (CDP-star; Thermo Fisher Scientific) and photographed with an LAS-4000 (GE Healthcare UK Ltd). The band intensities were scanned and quantified using the ImageJ software (http://rsb.info.nih.gov/ij/).

### Proteome profiler apoptosis array

To examine the expression of a panel of apoptosis-related molecules, cell lysates were analyzed using the Proteome Profiler^TM^ Human Apoptosis Array kit (R&D Systems) according to the manufacturer's protocol. In array analyses, samples of 500 μg protein lysate from cells were used. Densitometric analyses of array results were performed using the ImageJ software.

### Luciferase reporter assay

Panc-1 cells were transfected with control, HIF-1α, or HIF-2α siRNA using lipofectamine RNAiMAX. On the next day, the cells were additionally transfected with both TransLucent Survivin Gene Promoter Reporter Vector (Affymetrix) and pGL4.74(hRluc/TK) vector (Promega) using Lipofectamine 3000 according to the manufacturer's instructions. After 24 h incubation, firefly and Renilla luciferase activities were measured using the Dual-Glo Luciferase assay system (Promega), and the ratio of firefly/Renilla luciferase was determined.

### Chromatin immunoprecipitation (ChIP) assay

Crosslinked chromatin was prepared from siRNA-transfected Panc-1 cells, and ChIP assay was performed by using SimpleChIP Enzymatic Chromatin IP kit (CST) according to the manufacture's protocol. Antibody to HIF-2α (NOVUS) or normal rabbit IgG (CST) was used to precipitate the protein-chromatin complexes. Real-time PCR was performed using SYBR Premix Ex Taq II (TaKaRa Bio). The following primers (sense and anti-sense, respectively) were used: HRE-1, 5′-TTGAACTCCAGGACTCAAGTGAT-3′ and 5′-CCCCTCGACTGCTTTCAAAGAAC-3′ ; and HRE-2, 5′-GCGTTCTTTGAAAGCAGT -3′ and 5′-ATCTGGCGGTTAATGGCG-3′.

### *In vivo* xenograft mouse models

Female BALB nu/nu mice (6–7 weeks old) purchased from CLEA Japan (Tokyo, Japan) were maintained under specific-pathogen-free conditions. The protocols were approved by the Committee on the Ethics of Animal Experiments of the Shimane University Faculty of Medicine (Permit Number: IZ27-130, IZ27-132). All efforts were made to minimize suffering. In one experiment, nude mice were inoculated subcutaneously (s.c.) in the right flank at two different (upper and lower) sites with shRNA-expressing Panc-1 cells (5 × 10^6^) with Matrigel Matrix (Corning) at a 1:1 volume ratio in a total volume of 100 μL. When the tumor diameter reached approximately 5–6 mm, the mice were pooled and divided into four groups and injected with TRAIL (1 μg) intratumorally (i.t.) into the lower tumors at a volume of 50 μL for 5 consecutive days. As a vehicle control, the same volume of medium was injected i.t. into the upper tumors. In another experiment, the mice were inoculated s.c. in the right flank with Panc-1 cells (5 × 10^6^) with Matrigel. When the tumor diameter reached approximately 5–6 mm, they were pooled and divided into four groups and injected intraperitoneally (i.p.) with YM155 (5 mg/kg) on days 1, 2, 3, 4, and 5 after grouping and/or i.t. with TRAIL (1 μg) on days 3 and 5 after grouping. As a TRAIL vehicle control, the same volume of medium was injected. As a YM155 vehicle control, 100 μL DMSO was injected i.p. The tumor size was measured every 4 days. Each group contained six mice.

### Statistical analyses

Data were evaluated statistically using an unpaired two-tailed Student's *t*-test or ANOVA together with Bartlett's test. A *p*-value < 0.05 was considered to indicate statistical significance.

## SUPPLEMENTARY FIGURES



## References

[1] Ryan HE, Poloni M, McNulty W, Elson D, Gassmann M, Arbeit JM, Johnson RS (2000). Hypoxia-inducible factor-1alpha is a positive factor in solid tumor growth. Cancer Res.

[2] Palazon A, Aragones J, Morales-Kastresana A, de Landazuri MO, Melero I (2012). Molecular pathways: hypoxia response in immune cells fighting or promoting cancer. Clin Cancer Res.

[3] Kallio PJ, Wilson WJ, O’Brien S, Makino Y, Poellinger L (1999). Regulation of the hypoxia-inducible transcription factor 1alpha by the ubiquitin-proteasome pathway. J Biol Chem.

[4] Cramer T, Yamanishi Y, Clausen BE, Forester I, Pawlinski R, Mackman N, Haase VH, Jaenisch R, Corr M, Nizel V, Firestein GS, Gerber HP, Ferrara N (2003). HIF-1α is essential for myeloid cell-mediated inflammation. Cell.

[5] Hu CJ, Wang LY, Chodosh LA, Keith B, Simon MC (2003). Differential roles of hypoxia-inducible factor 1alpha (HIF-1α) and HIF-2α in hypoxic gene regulation. Mol Cell Biol.

[6] Talks KL, Turley H, Gatter KC, Maxwell PH, Pugh CW, Ratcliffe PJ, Harris AL (2000). The expression and distribution of the hypoxia-inducible factors HIF-1α and HIF-2α in normal human tissues, cancers, and tumor-associated macrophages. Am J Pathol.

[7] Krieg M, Haas R, Brauch H, Acker T, Flamme I, Plate KH (2000). Up-regulation of hypoxia-inducing factors HIF-1α and HIF-2α under normoxic conditions in renal carcinoma cells by von Hippel-Lindau tumor suppressor gene loss of function. Oncogene.

[8] Semenza GL (2010). Defining the role of hypoxia-inducing factor 1 in cancer biology and therapeutics. Oncogene.

[9] Zhao J, Du F, Shen G, Zhang F, Xu B (2015). The role of hypoxia-inducing factor-2 in digestive system cancers. Cell Death Dis.

[10] Takeda K, Yamaguchi N, Akiba H, Kojima Y, Hayakawa Y, Tanner JE, Sayers J, Seki N, Okumura K, Yagita H, Smyth MJ (2004). Induction of tumor-specific T cell immunity by anti-DR5 antibody therapy. J Exp Med.

[11] Zerafa N, Westwood JA, Cretney E, Mitchell S, Waring P, Iezzi M, Smyth MJ (2005). Cutting edge: TRAIL deficiency accelerates hematological malignancies. J Immunol.

[12] Kelley SK, Ashkenazi A (2004). Targeting death receptors in cancer with Apo2L/TRAIL. Curr Opin Pharmacol.

[13] Jin Z, McDonald ER, Dicker DT, El-Deiry WS (2004). Deficient tumor necrosis factor-related apoptosis-inducing ligand (TRAIL) death receptor transport to the cell surface in human colon cancer cells selected for resistance to TRAIL-induced apoptosis. J Biol Chem.

[14] Ng CP, Bonavida B (2002). X-linked inhibitor of apoptosis (XIAP) blocks Apo2 ligand/tumor necrosis factor-related apoptosis-inducing ligand-mediated apoptosis of prostate cancer cells in the presence of mitochondrial activation: sensitization by overexpression of second mitochondria-derived activator of caspase/direct IAP-binding protein with low pl (Smac/DIABLO). Mol Cancer Ther.

[15] Ng CP, Zisman A, Bonavida B (2002). Synergy is achieved by complementation with Apo2L/TRAIL and actinomycin D in Apo2L/TRAIL-mediated apoptosis of prostate cancer cells: role of XIAP in resistance. Prostate.

[16] Hari Y, Harashima N, Tajima Y, Harada M (2015). Bcl-xL inhibition by molecular-targeting drugs sensitizes human pancreatic cancer cells to TRAIL. Oncotarget.

[17] Giaccone G, Zatloukal P, Roubec J, Floor K, Musil J, Kuta M, van Klaveren RJ, Chaudhary S, Gunther A, Shamsili S (2009). Multicenter phase II trial of YM155, a small-molecule suppressor of survivin, in patients with advanced, refractory, non-small-cell lung cancer. J Clin Oncol.

[18] Satoh T, Okamoto I, Miyazami M, Morinaga R, Tsuya A, Hasegawa Y, Terashima M, Ueda S, Fukuoka M, Ariyoshi Y (2009). Phase I study of YM155, a novel survivin suppressant, in patients with advanced solid tumors. Clin Cancer Res.

[19] Rankin EB, Giaccia AJ (2008). The role of hypoxia-inducible factors in tumorigenesis. Cell Death Differ.

[20] Wilson WR, Hay MP (2011). Targeting hypoxia in cancer therapy. Nat Rev Cancer.

[21] Zhao J, Du F, Luo Shen YG, Zheng F, Xu B (2015). The emerging role of hypoxia-inducible factor-2 involved in chemo/radioresistance in solid tumors. Cancer Treat Rev.

[22] Zhao D, Zhai B, He C, Tan G, Jiang X, Pan S, Dong X, Wei Z, Ma L, Qiao H (2014). Upregulation of HIF-2α induced by sorafenib contributes to the resistance by activating the TGF-α/EGFR pathway in hepatocellular carcinoma cells. Cell Signal.

[23] Raval RR, Lau KW, Tran MG, Sowter HM, Mandriota SJ, Li JL, Pugh CW, Maxwell PH, Harris AL, Ratcliffe PJ (2005). Contrasting properties of hypoxia-inducible factor 1 (HIF-1) and HIF-2 in von Hippel-Lindau-associated renal cell carcinoma. Mol Cell Biol.

[24] Liu S, Kumar SM, Martin JS, Yang R, Xu X (2011). Snail1 mediates hypoxia-induced melanoma progression. Am J Pathol.

[25] Holmquist-Mengelbier L, Fredlund E, Lofstedt T, Noguera R, Navarro S, Nilsson H, Pietras HA, Vallon-Christersson J, Borg A, Gradin K, Poellinger L, Pahlman S (2006). Recruitment of HIF-1α and HIF-2α to common target genes is differentially regulated in neuroblastoma: HIF-2α promotes an aggressive phenotype. Cancer Cell.

[26] Mahajan S, Dammai V, Hsu T, Kraft AS (2008). Hypoxia-inducible factor-2α regulates the expression of TRAIL receptor DR5 in renal cancer cells. Carcinogenesis.

[27] Menrad H, Werno C, Schmid T, Copanaki E, Deller T, Dehne N, Brune B (2010). Roles of hypoxia-inducible factor-1α (HIF-1α) versus HIF-2α in the survival of hepatocellular tumor spheroids. Hepatology.

[28] Mayes PA, Dolloff NG, Daniel CJ, Liu JJ, Hart LS, Kuribayashi K, Allen JE, Jee DIH, Dorsey JF, Liu YY, Dicker DT, Brown JM, Furth EE (2011). Overcoming hypoxia-induced apoptotic resistance through combinatorial inhibition of GSK-3β and CDK1. Cancer Res.

[29] Jeong JK, Moon MH, Seo JS, Seol JW, Park SY, Lee YJ (2010). Hypoxia inducing factor -1α regulates tumor necrosis factor-related apoptosis-inducing ligand sensitivity in tumor cells exposed to hypoxia. Biochem Biophys Res Commun.

[30] Chen WC, Liu Q, Fu JX, Kang SY (2004). Expression of survivin and its significance in colorectal cancer. World J Gastroenterol.

[31] Colnaghi R, Connell CM, Barrett RM, Wheatley SP (2006). Separating the anti-apoptotic and mitotic roles of survivin. J Biol Chem.

[32] Altieri DC (2001). The molecular basis and potential role of survivin in cancer diagnosis and therapy. Trends Mol Med.

[33] Rodel F, Hoffmann J, Distel L, Herrmann M, Noisternig T, Papadopoulos T, Sauer R, Rodel C (2005). Survivin as a radioresistance factor, and prognostic and therapeutic target for radiotherapy in rectal cancer. Cancer Res.

[34] Schmitz M, Diestelkoetter P, Weigle B, Schmachtenberg F, Stevanovic S, Ockert D, Rammensee HG, Rieber EF (2000). Generation of survivin-specific CD8+ T effector cells by dendritic cells pulsed with protein or selected peptides. Cancer Res.

[35] Andersen MH, Pedersen LO, Becker JC, Straten PT (2001). Indentification of a cytotoxic T lymphocyte response to the apoptosis inhibitor protein survivin in cancer patients. Cancer Res.

[36] Tong WW, Tong GH, Chen XX, Zheng HC, Wang YZ (2015). HIF-2α is associated with poor prognosis and affects the expression levels of survivin and cyclin D1 in gastric carcinoma. Int J Oncol.

[37] Li W, Y Chen Y, Shen YB, Shu HM, Wang XJ, Zhao CL, Chen CJ (2013). HIF-1α knockdown by miRNA decreases surviving expression and inhibit A549 cell growth in vitro and in vivo. Int J Mol Med.

[38] Peng XH, Karna P, Cao Z, Jiang BH, Zhou M, Yang L (2006). Cross-talk between epidermal growth factor receptor and hypoxia-inducible factor-1α signal pathways increases resistance to apoptosis by up-regulating surviving gene expression. J Biol Chem.

[39] Hayashi N, Asano K, Suzuki H, Yamamoto T, Tanigawa N, Egawa S, Manome Y (2005). Adenoviral infection of survivin antisense sensitizes prostate cancer cells to etoposide in vivo. Prostate.

[40] Kunze D, Erdmann K, Froehner M, Wirth MP, Fuessel S (2012). siRNA-mediated inhibition of antiapoptotic genes enhances chemotherapy efficacy in bladder cancer cells. Anticancer Res.

[41] Nakahara T, Kita A, Yamanaka K, Mori M, Amino N, Takeuchi M, Tominaga F, Hatakeyama S, Kinoyama I, Matsuhisa A, Kudoh M, Sasamata M (2007). YM155, a novel small-molecule survivin suppressant, induces regression of established human hormone-refractory prostate tumor xenografts. Cancer Res.

[42] Yamanaka K, Nakahara T, Yamaguchi T, Kita A, Takeuchi M, Kiyonaga F, Kaneko N, Sasamata M (2011). Antitumor activity of YM155, a selective small-molecule survivin suppressant, alone and in combination with docetaxel in human malignant melanoma models. Clin Cancer Res.

[43] Kaneko N, Mitsuoka K, Amino N, Yamanaka K, Kita A, Mori M, Miyoshi S, Muromitsu S (2014). Combination of YM155, a survivin suppressant, with bendamustine and rituximab: a new combination therapy to treat relapsed/refractory diffuse large B-cell lymphoma. Clin Cancer Res.

[44] Rauch A, Hennig D, Schafer C, Wirth M, Marx C, Heinzel T, Schneider G, Kramer OH (2014). Survivin and YM155: how faithful is the liaison?. Biochem Biophys Acta.

[45] Shimojo Y, Akimoto M, Hisanaga T, Tanaka T, Tajima Y, Honma Y, Takenaga K (2013). Attenuation of reactive oxygen species by antioxidants suppresses hypoxia-induced epithelial-mesenchymal transition and metastasis of pancreatic cancer cells. Clin Exp Metastasis.

