# Supplementary material for: HIF-2α dictates the susceptibility of pancreatic cancer cells to TRAIL by regulating survivin expression
Source: Oncotarget. 2017 Apr 17;8(26):42887–900. doi: 10.18632/oncotarget.17157 (PMC5522113; doi:10.18632/oncotarget.17157)
Supplement: Supplementary file 1 [file oncotarget-08-42887-s001.pdf]

HIF-2α dictates the susceptibility of pancreatic cancer cells to TRAIL by regulating survivin expression

SUPPLEMENTARY MATERIALS

SUPPLEMENTARY FIGURES

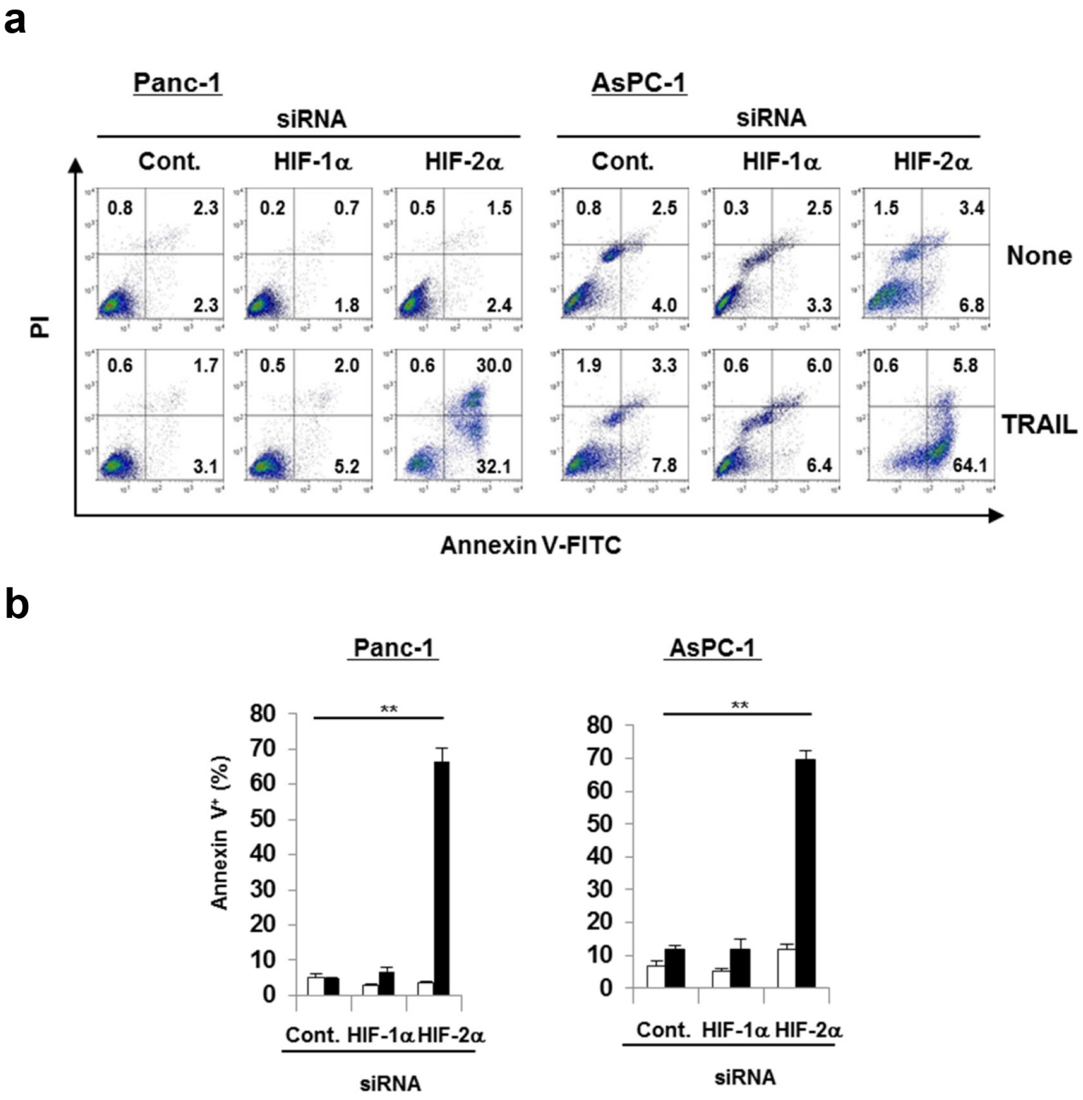

Supplementary Figure 1: Transfection of HIF-2α siRNA increases TRAIL sensitivity of pancreatic cancer cells. (a) siRNA-transfected Panc-1 and AsPC-1 cells were incubated with TRAIL (100 ng/mL for 24 h for Panc-1) or (50 ng/mL, 12 h for AsPC-1) under hypoxic conditions and flow cytometric analysis was performed after staining with FITC-conjugated Annexin V and PI. **(b)** The results are presented as means ± SD from triplicate experiments. The open and closed bars represent the data of control and TRAIL treatment, respectively. ** $p < 0.01$.

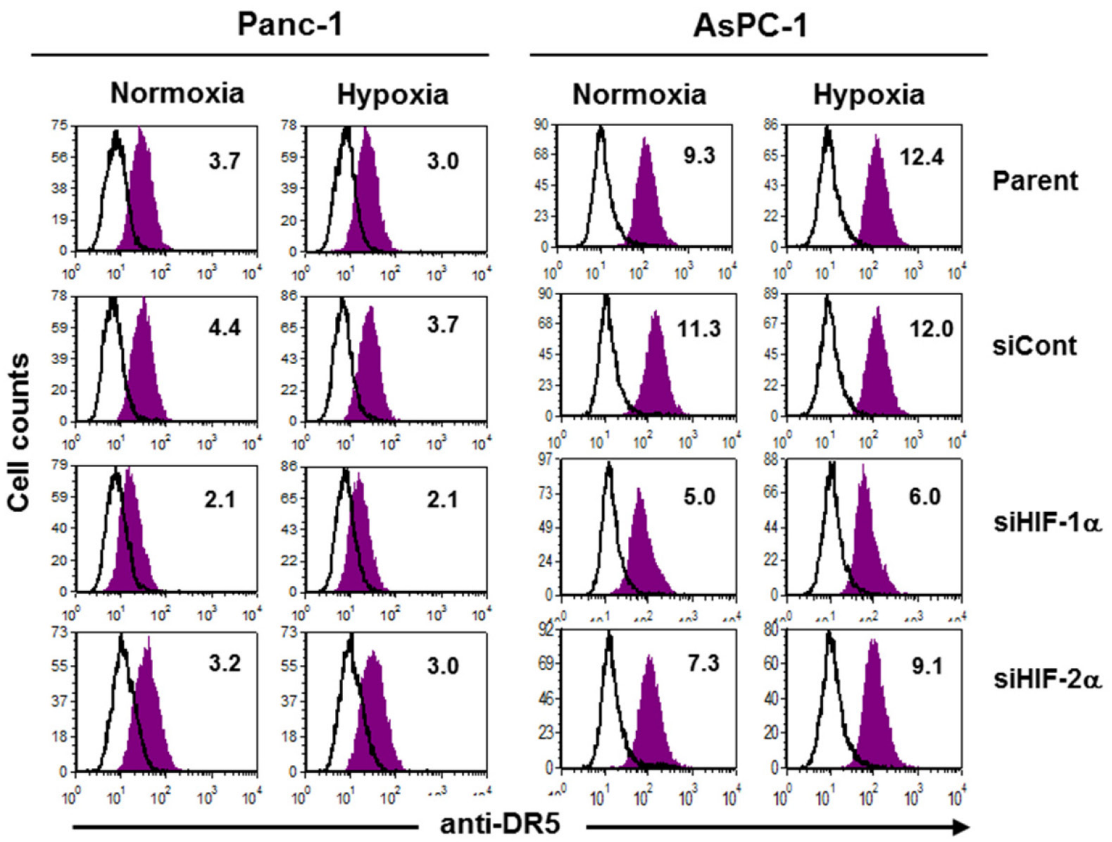

Supplementary Figure 2: DR5 expression on Panc-1 and AsPC-1 cells. Panc-1 and AsPC-1 cells pre-transfected with control, HIF-1α, or HIF-2α siRNA were stained with anti-DR5 antibody or isotype-matched IgG, followed by FITC-conjugated anti-mouse IgG. The open and closed backgrounds are the staining with either isotype-matched IgG or anti-DR5 antibody, respectively. The numbers represent the ratio of fold change. Fold change = mean fluorescence intensity (MFI) of anti-DR5 Ab/MFI of isotype-matched control.

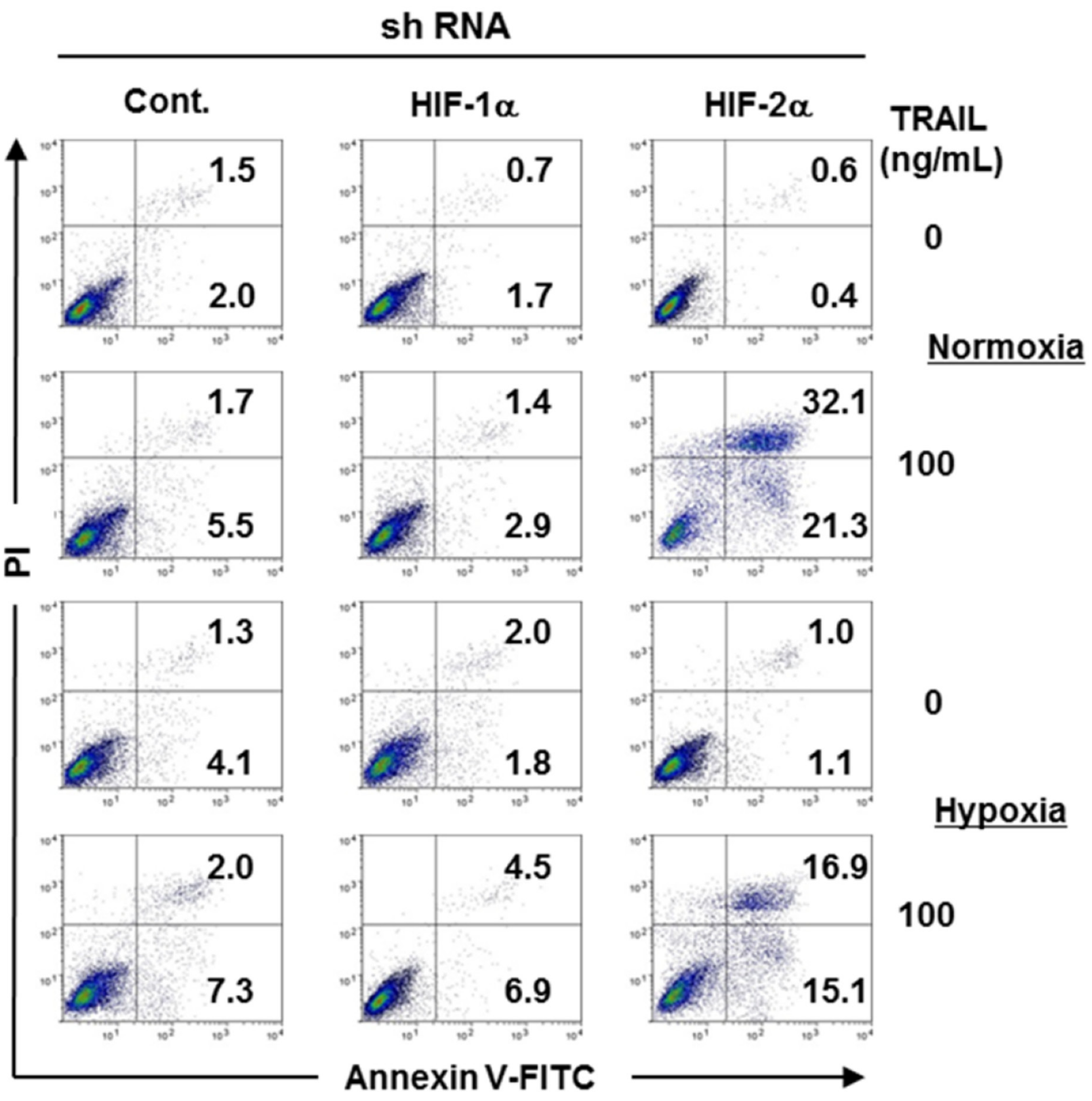

Supplementary Figure 3: Sensitivity of shRNA-expressing Panc-1 cells to TRAIL. shRNA-expressing Panc-1 cells were treated with TRAIL under normoxic or hypoxic (1% O2) condition for 24 h and flow cytometric analysis was performed after staining with Annexin V-FITC and PI. The numbers represent the percentage of each subset.

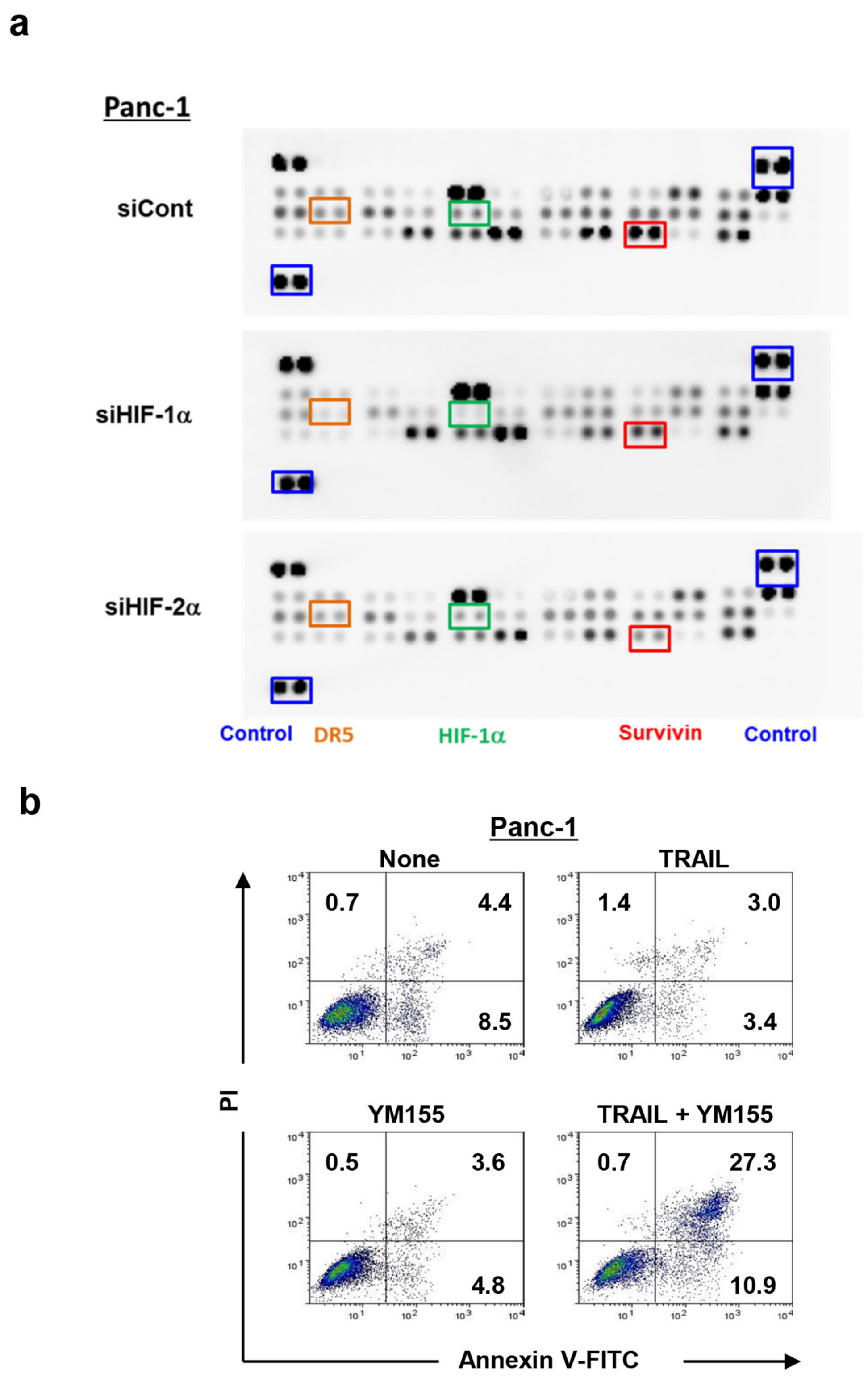

Supplementary Figure 4: Array assay of apoptosis-related proteins and effects of a combination of TRAIL and YM155 on apoptosis of Panc-1 cells. **(a)** The results of Proteome Profiler Human Apoptosis Array on siRNA-transfected Panc-1 cells are shown. The assay was performed in duplicate. The protein dotes of survivin, HIF-1α, DR, and the control are marked. **(b)** Panc-1 cells were cultured with TRAIL (25 ng/mL) and /or YM155 (5 nM) for 24 h. Thereafter, flow cytometric analysis was performed after staining with Annexin V-FITC and PI. The numbers represent the percentage of each subset.
